# Cross-education: motor unit adaptations mediate the strength increase in non-trained muscles following 8 weeks of unilateral resistance training

**DOI:** 10.3389/fphys.2024.1512309

**Published:** 2025-01-07

**Authors:** Edoardo Lecce, Alessandra Conti, Alessandro Del Vecchio, Francesco Felici, Alessandro Scotto di Palumbo, Massimo Sacchetti, Ilenia Bazzucchi

**Affiliations:** ^1^ Laboratory of Exercise Physiology, Department of Movement, Human, and Health Sciences, University of “Foro Italico, Rome, Italy; ^2^ Department Artificial Intelligence in Biomedical Engineering, Faculty of Engineering, Zentralinstitut für Medizintechnik (ZIMT), Friedrich-Alexander University Erlangen-Nürnberg, Erlangen, Germany

**Keywords:** cross-education, EMG, motor unit, neuromuscular adaptations, resistance training, strength

## Abstract

**Introduction:**

Early increases in muscle strength following unilateral resistance training are typically accompanied by strength gains in the contralateral untrained muscles, a phenomenon known as cross-education. However, the specific motor unit adaptations responsible for this gain transfer remain poorly understood. To address this gap, we recorded myoelectrical activity from the biceps brachii using high-density electromyography.

**Methods:**

Nine participants performed 8-week unilateral resistance training and were compared to nine control individuals who did no intervention. Discharge characteristics of longitudinally tracked motor units were assessed during maximal voluntary contractions and isometric ramp contractions at 35% and 70% of the maximal voluntary force (MVF) at baseline (T0), 4 weeks (T1), and 8 weeks (T2) post-intervention.

**Results:**

MVF increased by 7% in untrained muscles at T1 and 10% at T2 (p < 0.05). These gains were accompanied by significant decreases in motor unit recruitment thresholds (p < 0.01) and higher net discharge rate (i.e., gain in discharge rate from recruitment to peak) following intervention (p < 0.05). Trained muscles presented greater MVF (+11%, T1; +19%, T2) with similar motor unit adaptations, including a lower recruitment threshold (p < 0.01) and a higher net discharge rate (p < 0.01).

**Discussion:**

Our findings indicate that higher strength in untrained muscles is associated with a higher net discharge rate, implying a greater spinal motoneuron output to muscles. The present results underscore the role of motor unit adaptations in the transfer of strength gains to non-trained muscles, offering novel insights into the neural mechanisms underlying cross-education.

## 1 Introduction

Resistance training is a central component in developing and supporting motor functionality by promoting hypertrophy, muscle strength, and power increase (Kramer et al., 2002; Carr et al., 2019; Pearcey et al., 2021). These improvements result from a combination of neural and morphological adaptations in response to mechanical overload applied to the neuromuscular system (Folland and Williams, 2007). Initial gains are typically evident after 4 weeks of resistance training (Del Vecchio et al., 2019a; Lecce et al., 2024b) and primarily attributed to neural modifications (Sale, 1988; Del Vecchio et al., 2024), including motor unit adaptations either independently or in conjunction with morphological changes (Duchateau et al., 2006; Ansdell et al., 2020; Škarabot et al., 2021; Roberts et al., 2023). A typical instance of such early neural changes is *cross-education*, also known as *cross-transfer* or *interlimb transfer* (Manca et al., 2018), in which unilateral resistance training induces gain transfers to the contralateral untrained limb (Moritani and deVries, 1979; Manca et al., 2021; Kay et al., 2024). These gains seem specific to strength and skill transfer, as muscle endurance does not seem to transfer to the untrained side (Song et al., 2024).

Following relatively short-term unilateral resistance training (∼4 weeks), untrained muscles typically exhibit an average strength increase of 7.6%, approximately half the gain observed in the trained side (Hendy and Lamon, 2017). Specifically, improvements in untrained muscles vary from 6% to 10% in the upper limbs and 13%–16% in the lower limbs, depending on the type of intervention and its duration (Latella et al., 2012; Hendy and Lamon, 2017; Green and Gabriel, 2018; Manca et al., 2018). Notably, 8 weeks of eccentric resistance training has been shown to induce strength transfers of up to 20% in upper limbs (Howatson et al., 2011; Boyes et al., 2017; Green and Gabriel, 2018; Martinez et al., 2021; Sato et al., 2021) with effects lasting more than those observed with concentric contractions (Harput et al., 2018). Despite cross-education being known for over a century (Scripture et al., 1894), the precise physiological mechanisms underlying this phenomenon remain a subject of ongoing investigation and debate (Barss et al., 2016; Manca et al., 2018; Paillard, 2020).

According to the existing evidence, cross-education is thought to be mediated by adaptations within the central nervous system, particularly involving motor cortices (Frazer et al., 2018). The bilateral access and cross-activation models offer complementary views at the central level. The bilateral access model suggests that a shared motor engram, accessible by both cortices via the corpus callosum, enables cross-limb performance improvements without concurrent bilateral cortical activation. In contrast, the cross-activation model proposes that unilateral training leads to simultaneous activation of both motor cortices, facilitating interhemispheric communication through reduced inter-hemispheric inhibition (IHI) (Ruddy and Carson, 2013; Richmond and Fling, 2019). Furthermore, the bilateral interaction may occur through uncrossed corticofugal fibers failing to decussate at the pyramidal level, together with branched bilateral corticomotoneuronal projections innervating contralateral homologous motor pools (Carson, 2005; Leung et al., 2018). Reduced IHI enhances connectivity between hemispheres, while decreased short-interval intracortical inhibition in the untrained hemisphere increases cortical excitability, supporting neural adaptations (Hortobágyi et al., 2011).

These cortical changes have potential downstream effects at the spinal level, as indicated by increased signal amplitude in untrained muscles (Lee and Carroll, 2007; Pelet and Orsatti, 2021) and increased V-wave, suggesting enhanced neural drive to the muscles (Fimland et al., 2009). A greater H_max_/M_max_ ratio has also been reported, implying greater spinal excitability (Bouguetoch et al., 2021). These adaptations have been hypothesized to contribute to motor unit recruitment and firing modifications, revealing how both cortical and spinal mechanisms contribute to the neural plasticity of cross-education (Latella et al., 2012; Bundy and Leuthardt, 2019). Nevertheless, no modifications in motor unit discharge rate have been observed, a finding attributed to methodological limitations in motor unit exploration (Green and Gabriel, 2018). These limitations could be overcome by employing higher spatial resolution electromyography (HDsEMG) and advanced tracking approaches (Holobar et al., 2014; Martinez-Valdes et al., 2017; Del Vecchio et al., 2020).

Although HDsEMG has been demonstrated to be a reliable tool in assessing motor unit response to resistance training (Del Vecchio et al., 2019a; Elgueta-Cancino et al., 2022; Orssatto et al., 2023), no studies have yet explored physiological adaptations underlying cross-education by examining longitudinally tracked motor units. To address this gap, we recorded myoelectrical activity with HDsEMG from biceps brachii (BB), as it is recognized as the primary muscle responsible for elbow flexion force (Dartnall et al., 2008; Yu et al., 2022). This muscle has also been extensively assessed in cross-education studies, demonstrating significant adaptations in the untrained limb (Farthing and Chilibeck, 2003; Manca et al., 2018). We aimed to investigate changes in discharge characteristics of longitudinally tracked motor units in response to 8 weeks of unilateral resistance training to understand the mechanisms underlying the increase in strength of the untrained limb.

Based on the abovementioned evidence, we expected to observe (a) higher muscle strength, (b) lower motor unit recruitment thresholds, and (c) augmented discharge rate driving greater force levels in both trained and untrained limbs. Additionally, (d) lower motor unit firing variability, an adaptation associated with increased strength, was also hypothesized (Vila-Chã and Falla, 2016).

## 2 Methods

### 2.1 Participants and ethical approval

The study was approved by the local ethical committee of the University of ‘Foro Italico,’ Rome (approval n. CAR157/2023) and conformed to the *Declaration of Helsinki* standards. All the participants signed a written informed consent form explaining the experimental procedures and potential side effects in detail before enrolling, underlining that they could withdraw from the protocol at any time without jeopardy. Twenty recreationally active participants (males, n = 10; females, n = 10) were initially enrolled, but two withdrew from the study. Therefore, eighteen participants (males, n = 8; females, n = 10) began and concluded the 8-week training protocol. Following familiarization, volunteers were randomly assigned to either intervention (INT, n = 9; females, n = 5) or control (CNT, n = 9; females, n = 5) group by adopting the block-randomization approach, ensuring an equal number per group (Kang et al., 2008). They were assigned unique alphanumeric codes to ensure their privacy and confidentiality. Participants with metabolic disease, upper limb musculoskeletal disorders, acute infection, uncontrolled hypertension, those under medications that impact muscle protein metabolism, modulate vascular tone and neural activity, and those using contraceptives (Burrows and Peters, 2007; Elliott-Sale et al., 2020; Reif et al., 2021) were excluded. Inclusion criteria included an age between 18 and 35 years and good health. Participants’ anthropometric characteristics are reported in Table 1.

**TABLE 1:** Anthropometric comparisons at baseline.

	Group		
	INT (n = 9)	CNT (n = 9)	*p*-values
Age (years)	24.4 ± 1.6	24.5 ± 1.6	0.891
Height (m)	1.67 ± 0.11	1.72 ± 0.07	0.190
Mass (kg)	61.8 ± 19.1	69.9 ± 19.1	0.269
BMI (kg⋅m^-2^)	21.8 ± 2.5	23.1 ± 4.8	0.464

### 2.2 Overview of the study

Participants were asked to be available for four laboratory visits, the first for familiarization and the others for the neuromuscular tests at the baseline (T0), at the fifth week (T1), and the 10th week (T2). Volunteers from the intervention group were asked to perform 24 training sessions in the laboratory, split into 12 between T0 and T1 and 12 between T1 and T2, with a total of 8 weeks of training. Five to 7 days of wash-out prior to the neuromuscular tests were assured to minimize muscle soreness and training-lasting effects (Amstrong, 1984; Newham, 1988; Mizumura and Taguchi, 2024). During the first visit, volunteers received information concerning the experimental and testing procedures and were familiarized with maximal voluntary isometric contractions (MVICs) and trapezoidal isometric contractions (ramps) of the biceps brachii to ensure reliable performance of task and proper steady phase execution. The dominant limbs were identified by employing the Edinburgh Questionnaire (Oldfield, 1971). No measurements were done during this first visit. Data collection started during the second visit (T0), including the identification of the maximal voluntary force (MVF), submaximal ramp contractions, and HDsEMG recordings from the biceps brachii of both limbs. In addition, arm circumferences and subcutaneous skinfold thickness (SST) were assessed to account for changes in muscle size (Mei et al., 2002). According to previous findings, female participants completed the test during either the ovulatory or mid-luteal phase to minimize fluctuations in neuromuscular activity and avoid the general decrease in activation observed during the early follicular phase (Tenan et al., 2013; Weidauer et al., 2020). Therefore, the test was repeated in the same phase as the baseline (Piasecki et al., 2024).

### 2.3 Experimental procedures

Participants were asked to refrain from strenuous exercise and caffeine consumption 48 h before the tests. The testing procedures were performed after a standardized warm-up, consisting of 3 x 30 isometric BB contractions at 30%–40% MVF, separated by 30 s. They were asked to focus on elbow flexion and to, as much as possible, isolate the muscle contraction of the biceps brachii during the warm-up and tests. Consequently, the participants performed three MVICs with 180 s of rest between trials, and they were asked to push as hard as possible for 5 s while receiving verbal encouragement to achieve a higher peak for each contraction. The MVF was set as the greatest value recorded across the three MVICs and was used to set the submaximal ramp contraction target forces. After 5 min from the last maximal trial, participants performed a ramp contraction per target force (35% or 70% MVF) in a randomized order and separated by 5 min. Ramp contractions consisted of a linear force increase (ramp-up) at a rate of 5% MVF s^-1^ to a target value, which was maintained for 10 s, and a linear force decrease (ramp-down) back to the resting value at the same rate as the increasing phase. Participants received visually guided feedback on their force trajectory via a monitor positioned 1.5 m away. For each testing session, the target force for ramp contractions was set as a percentage of the maximal voluntary force measured in the same testing session, ensuring that the target force was always based on the daily MVF.

### 2.4 Training protocol

**FIGURE 1:**
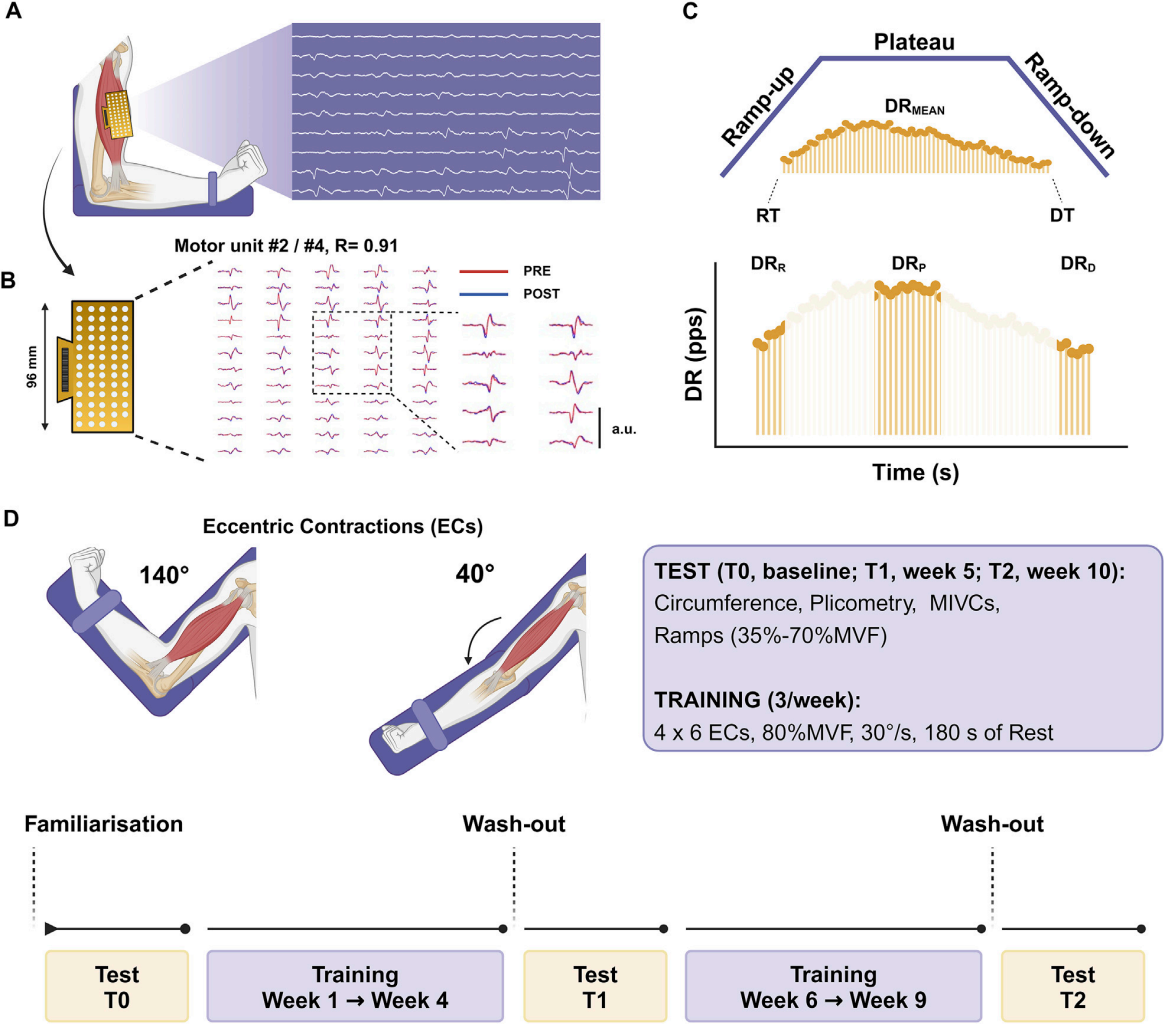
Experimental setup **(A)** The arm was placed on a dynamometer at 90° of elbow flexion with HDsEMG grids recording the signals. **(B)** Myoelectrical signals were decomposed into individual motor unit discharge patterns, and the tracking procedure was used to identify the same motor units across the testing sessions during the offline analysis [representative example: identified MU #2 at T0 showed a positive association with identified MU #4 at T1, r = 0.91]. **(C)** Trapezoidal isometric contractions were adopted to assess individual motor unit characteristics by identifying the recruitment and derecruitment thresholds (RT and DT), the averaged discharge rate (DR_MEAN_) and the discharge rate at recruitment (DR_R_), plateau (DR_P_), and derecruitment (DR_D_). **(D)** Testing sessions were repeated at baseline (T0), after 4 weeks (T1), and after 8 weeks (T2). The 8-week training protocol was split into two periods lasting 4 weeks each and included eccentric contractions at 80%MVF, which refers to the baseline in the first stage and to T1 in the second stage. The image was created with Biorender.

The intervention group performed unilateral eccentric strength training of non-dominant limbs three times per week in an 8-week training period divided into 4 weeks between T0 and T1 and the remaining 4 weeks between T1 and T2 (Figure 1). The eccentric protocol consisted of a standardized warm-up of 3 × 10 dynamic contractions at 30% MVF (separated by 60 s), and 2 × 4 eccentric contraction at 50% MVF (separated by 120 s). This was followed by four sets of six elbow-flexor eccentric contractions at 30°/s (from 140° to 40° of flexion) at 80% MVF (separated by 180 s). The non-dominant limb was chosen for training, as suggested by previous evidence (Farthing et al., 2007).

### 2.5 Force signal recording

The elbow-flexion force was evaluated with an isokinetic dynamometer (Kin-Com, Chattanooga, Tennessee). Participants were seated in the dynamometric chair and stabilized by chest and waist straps. The upper arm was parallel to the trunk, with the forearm halfway between supination and pronation, with an elbow flexion of 90° (Figure 1). The lever arm center of rotation was aligned with the distal lateral humerus epicondyle, and the wrist was secured in a cuff attached to the load cell. The analog force signal was sampled and amplified at 2048 Hz with an external analog-to-digital (A/D) converter (EMG-400, OT-Bioelettronica, Turin, Italy) and synchronized with the electromyogram. Since ramp contractions needed to be visually guided, a trapezoidal pattern was shown to participants during the contraction. A minimum/maximum error margin of 3% MVF was displayed along the entire trapezoidal force trajectory, which represented the target force value for participants to follow. This interval was designed to help participants remain within the specified margin of error.

### 2.6 High-density surface electromyography recordings (HDsEMG)

HDsEMG signals were recorded from the BB one limb at a time with an adhesive grid of 64 electrodes [13 rows x five columns; gold-coated; diameter: 1 mm; inter-electrode distance (IED): 8 mm; OT-Bioelettronica, Turin, Italy]. After skin preparation (shaving, light skin abrasion, and 70% ethanol cleansing), the muscle perimeter was identified through palpation and marked with a surgical pen. The grid orientation was based on recordings from a 16-electrode array (IED 5 mm, OT-Bioelettronica, Turin, Italy), and the innervation zone (IZ) was identified to estimate the fiber direction, as previously described (Lecce et al., 2023b). The IZ was located by identifying the inversion point in the action potential propagation direction proximally (toward the BB proximal tendon) and distally (toward the BB distal tendons) along the electrode column. The grid was placed right over the IZ (Huang et al., 2019) over the muscle belly, using a disposable biadhesive with layer holes adapted to the HDsEMG grids (SpesMedica, Battipaglia, Italy). The foam layer holes were filled with a conductive paste (SpesMedica, Battipaglia, Italy) to ensure skin-electrode contact. Reference electrodes were positioned on the ulna styloid process, the acromion skin surface, and the medial malleolus. HDsEMG signals were recorded in monopolar mode and converted to digital data by a 16-bit multichannel amplifier (EMG-Quattrocento, OT Bioelettronica, Turin, Italy), amplified (x150), sampled at 2048 Hz, and band-pass filtered (10–500 Hz) before being stored for offline analysis.

### 2.7 Force and HDsEMG analysis

The force signal was converted to newtons (N), and gravity compensation was used to remove the offset. The signal was low-pass filtered with a fourth-order, zero-lag Butterworth filter with a cut-off frequency of 15 Hz. Trapezoidal contractions presenting countermovement action or pre-tension were discarded from the analysis (Lecce et al., 2023a). Before decomposing into individual motor unit action potentials, the monopolar EMG recordings were band-pass filtered at 20–500 Hz (second-order, Butterworth). The raw HDsEMG signals were decomposed using the convolutive blind source separation method (Holobar and Zazula, 2007) implemented in the DEMUSE software working in MATLAB (MathWorks Inc. Natick, United States). This decomposition procedure can identify motor unit discharge times over a broad range of forces (Holobar and Farina, 2014). An experienced investigator manually analyzed all the identified motor units and retained only those characterized by a high pulse-to-noise ratio [>30 dB] (Holobar et al., 2014). Briefly, the manual analysis consists of inspection and editing procedures after automatic decomposition, involving the examination of motor unit spike trains during the whole contraction, discarding those motor units with a pulse-to-noise ratio below the reference threshold [30 dB] for accuracy (Del Vecchio et al., 2020; Hug et al., 2021).

The recruitment [RT] and derecruitment thresholds [DT] were identified as the absolute (N) and the relative force (% MVF) at which motor units were activated and deactivated, identifying the first and the last spikes, respectively (Del Vecchio et al., 2019a). The average discharge rate was computed from the series of discharge times identified by the automatic decomposition, providing the discharge number per second for each motor unit across the whole activity time (DR_MEAN_). This parameter was related to the average neural drive (cumulative neural activity) of motoneurons (Del Vecchio et al., 2019b; Del Vecchio et al., 2024). The discharge rate for each motor unit was also assessed during the recruitment (i.e., the average of the first four action potentials, DR_R_), during the plateau (i.e., the average of the first 10 action potentials, DR_P_), and at derecruitment (i.e., the average of the last four action potentials, DR_D_). The *net discharge rate* (Net-DR) was calculated as the gain in the discharge rate from motor unit recruitment to the target force of ramp contractions, as previously indicated (Del Vecchio et al., 2019a; Nuccio et al., 2021; Lecce et al., 2024a).

The interspike interval variability (ISIv) of motor units and the coefficient of variation of force (CovF), defined as the percent ratio between the standard deviation and the mean force ([SD/mean]x100), were calculated over the central 8 s of the plateau phase of ramp contractions discarding the first and the last 1-s period to minimize fluctuations due to the ramp-up/ramp-down transitions. The association between these two variables was also computed to account for the change in force steadiness relative to firing variability (Tracy et al., 2005; Enoka and Farina, 2021). The input-output gain of motoneurons was additionally estimated by computing the association between the change in motor unit discharge rate (∆-DR) and the relative force exerted (∆-Force) during the ramp-up phase of contractions. This analysis estimates the input-output gain of the motoneurons as it reflects the extent of the gain in motor unit discharge rate from the activation to the target force (i.e., Net-DR) relative to the change in exerted force. A change in this association indicates a modification in the motoneuronal output to a certain level of synaptic input when the system requires to exert force (Del Vecchio et al., 2019a; Ferguson and Cardin, 2020; Casolo et al., 2021; Lecce et al., 2024).

Motor units were longitudinally tracked across the intervention (T0, T1, and T2) to ensure the reliability of motor unit comparisons. This technique is based on the correlation value of the two-dimensional action potential waveforms (Martinez-Valdes et al., 2017). Only motor units with a high correlation value were considered for the analysis (arbitrary R > 0.8). Since our setup included three timelines, the percentage of tracked motor units drastically decreased as we performed a first tracking procedure (T0 - T1) and a second procedure (T1 - T2), considering only those motor units matched at the first stage. Tracking procedures were performed between the same contraction intensity trials (e.g., 35% MVF: T0 - T1) for measurement reliability.

### 2.8 Statistical analysis

The Shapiro-Wilk test was used to assess the data distribution normality. Between-group comparisons were performed at baseline using independent sample t-tests for the average number of identified and tracked MUs, SST, and MVF. The average number of identified motor units, SST, CovF at 35% and 70% MVF, MVF, and the ∆MVF, were compared with paired samples t-tests to account for the within-participant dependence in PRE-POST intervention evaluations. Differences in the DR_MEAN_, DR_R_, DR_P_, DR_D_, Net-DR, ISIv, RT, and DT across the timelines for each group were assessed using mixed-effect linear regression analysis to incorporate the whole sample of extracted motor units, which preserves variability within and across participants simultaneously to the greatest extent (Wilkinson et al., 2023). Group, limb, time and the interaction between these were considered fixed effects, with a random intercept for each participant [e.g., discharge rate ∼ group x limb x time + (1 | Participant ID)]. Bonferroni corrections were conducted in case of significant interaction, and the estimated marginal means with 95% confidence intervals between pre and post-intervention were computed. The association between the change in the discharge rate and force during the ramp-up phase of contraction was computed as the analysis of the input-output gain of motoneurons (Del Vecchio et al., 2019a; Casolo et al., 2021). In order to examine whether changes in the maximal voluntary force (∆-MVF) were associated with the change in the average *net discharge rate* (∆-Net-DR), linear regression analyses were performed between these variables. The coefficient of determination was also computed to assess the strength of the association for the following variables: a) the ∆MVF in trained and untrained limbs; b) the mean ISIv and CovF. Slope comparisons were performed using ANOVAs (Andrade and Estévez-Pérez, 2014). Cohen’s d was used as the effect size for significant results in the t-tests. To estimate the proportion of variance a specific predictor explains while controlling for random effects, partial eta squared was computed for significant results of mixed-effect analysis as previously suggested (Correll et al., 2022). The statistical calculations were done using jamovi 2.3.18 (The jamovi project, Sydney, Australia) and SPSS 25.0 (IBM Corp., Armonk, NY, United States). A *p* < 0.05 was considered a statistically significant result. Data are reported as the mean ± SD in the text.

## 3 Results

Anthropometric data of both INT and CNT groups are reported in Table 1. A complete statistical report, including results for both the intervention and control groups, is uploaded as supplementary material.

### 3.1 MVF, CovF, SST, and limb circumferences

Between-group comparisons at baseline revealed similar MVF of both the dominant (INT, 260 ± 107 N vs. CNT, 266 ± 99 N; *p* = 0.421) and non-dominant (INT, 244 ± 92 N vs. CNT, 250 ± 93 N; *p* = 0.393) limbs. Similarly, no differences were observed comparing SST of the dominant (INT, 4.5 ± 2.3 mm vs. CNT, 5.1 ± 2.2 mm; *p* = 0.217) and non-dominant (INT, 5.2 ± 2.3 mm vs. CNT, 5.9 ± 2.9 mm; *p* = 0.294) limbs. In addition, no differences were found in limb circumference between-group comparisons of the dominant (INT, 27.6 ± 3.7 cm vs. CNT, 28.1 ± 4.0 cm; *p* = 0.259) and non-dominant (INT, 27.5 ± 3.3 cm vs. CNT, 27.8 ± 4.0 cm; *p* = 0.394) sides.

**FIGURE 2:**
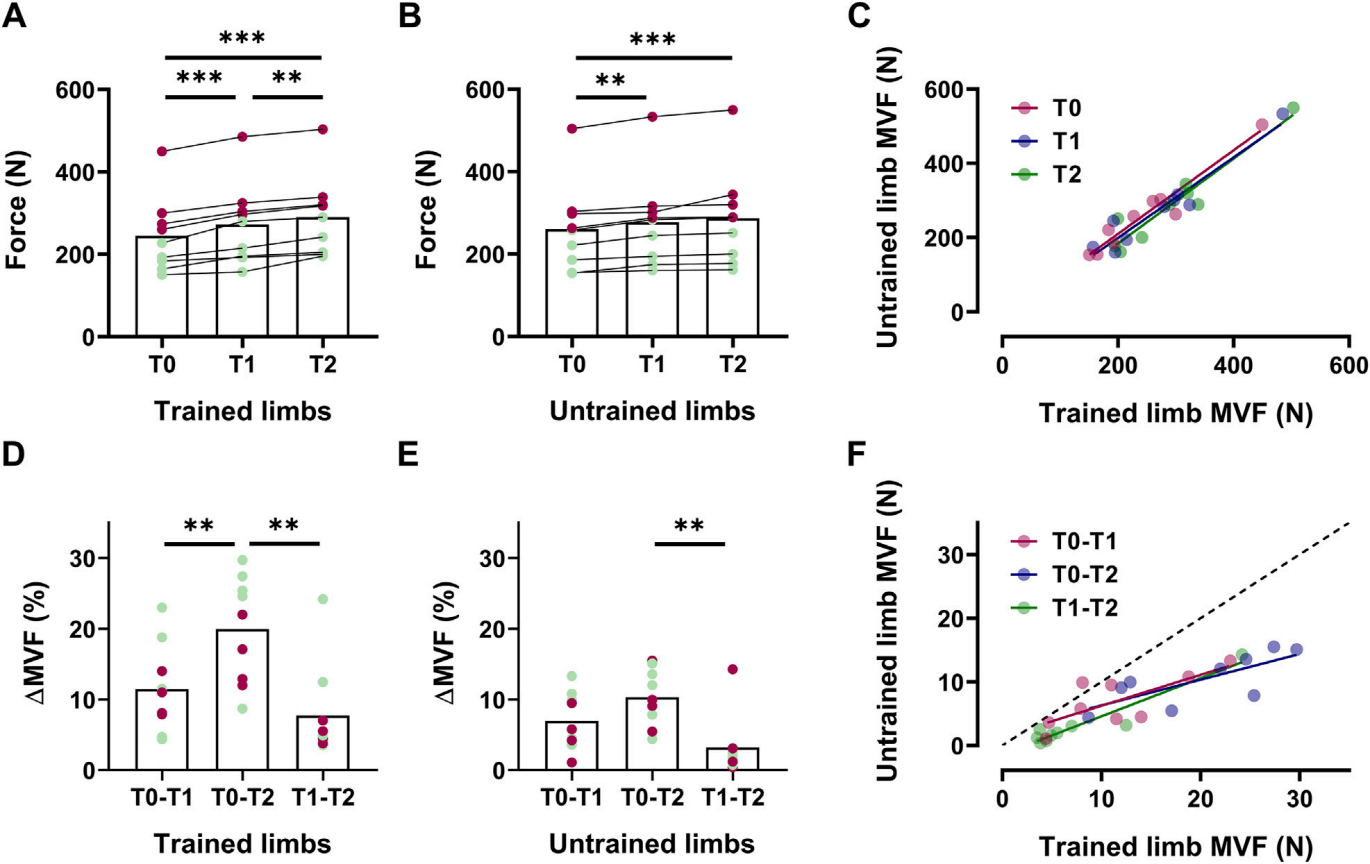
The increase in the MVF in trained and untrained limbs Box plots with individual markers for the absolute values of the maximal voluntary force (MVF) across intervention for trained **(A)** and untrained **(B)** limbs, with a scatter plot displaying the association between these values **(C)**. Delta (%) of the increase in MVF reported for trained **(D)** and untrained **(E)** sides between each timeline, with the association between these values as a scatter plot **(F)**. In box plots, red markers represent male individuals, whereas green markers represent female individuals.

In the intervention group, trained limbs presented an increase in MVF of 11% at T1 [∆MVF = +28 N [14, 41], *p* < 0.001, d = 1.93 (Figure 2A)] and 19% at T2 [∆MVF = +46 N [33, 58], *p* < 0.001, d = 3.38 (Figure 2A)]. A statistically significant difference was also observed between T1 and T2, with an increase of approximately 7% [∆MVF = +18 N [5, 31], *p* < 0.001, d = 1.86 (Figure 2A)]. In the untrained muscle, MVF increased by 7% at T1 [∆MVF = +17 N [2, 32], *p* = 0.001, d = 1.76 (Figure 2B)] and 10% at T2 [∆MVF = +26 N [10, 42], *p* < 0.001, d = 2.00 (Figure 2B)], with no significant differences between T1 and T2 (*p* = 0.107).

We found statistically significant associations between MVF of trained and untrained limbs T0 (*R* ^2^ = 0.93, *p* < 0.001), T1 (*R* ^2^ = 0.92, *p* < 0.001), and T2 (*R* ^2^ = 0.90, *p* < 0.001) (Figure 2C). In trained limbs, we observed significant differences in ∆MVF when comparing the increase observed in 8 weeks (T0-T2) compared to the two individual interventions separately [T0-T1, ∆MVF = +8.5% [3.1, 13.9], *p* = 0.007, d = 1.21; T1-T2, ∆MVF = +12.2% [7.3, 17.2], *p* < 0.001, d = 1.90 (Figure 2D)]. In contrast, untrained limbs only present a significant difference comparing 8 weeks (T0-T2) to the last 4 weeks of intervention [T1-T2, ∆MVF = +7.1% [4.0, 10.2], *p* < 0.001, d = 1.21 (Figure 2E)], confirming that the magnitude of increase from the fourth to the eighth week was not significant. Nevertheless, the association between ∆MVF was statistically significant across all the timelines [*p* < 0.01 (Figure 2F)].

Trained limbs presented a significant decrease in the CovF-35% at T1 (∆CovF = −0.66% [-0.74, −0.58], *p* = 0.03, d = 1.07) and T2 (∆CovF = −1.16% [-1.31, −1.01], *p* = 0.001, d = 2.08), with significant differences between the fourth and the eighth week (∆CovF = −0.50% [-0.66, −0.34], *p* = 0.04, d = 0.87). Similarly, CovF also decreased at 70% MVF for trained limbs at T1 (∆CovF = −0.45% [-0.53, −0.37], *p* = 0.03, d = 0.71) and T2 (∆CovF = −0.95% [-1.12, −0.78], *p* < 0.001, d = 1.59), with significant differences between T1 and T2 (∆CovF = −0.50% [-0.68, −0.32], *p* = 0.02, d = 0.83). Compared to the baseline, untrained limbs exhibited significantly lower CovF at 35% MVF at T1 (∆CovF = −0.58% [-0.69, −0.47], *p* = 0.03, d = 1.03) and T2 (∆CovF = −0.69% [-0.80, −0.58], *p* = 0.008, d = 1.22), with no significant differences between T1 and T2 (*p* = 0.150). CovF also declined at 70% MVF in the untrained limbs at T1 (∆CovF = −0.30% [-0.39, −0.21], *p* = 0.03, d = 0.51), T2 (∆CovF = −0.53% [-0.62, −0.44], *p* = 0.003, d = 0.83), with no significant differences between T1 and T2 (*p* = 0.163).

In trained limbs, the SST significantly decreased at T1 [∆SST = −0.58 mm [-0.76, −0.39], *p* = 0.04, d = 0.28 (Table 2)] and T2 [∆SST = −0.80 mm [-0.96, −0.63], *p* = 0.01, d = 0.36 (Table 2)]. Significant differences were also observed between T1 and T2 for the same parameter [∆SST = −0.22 mm [-0.36, −0.07], *p* = 0.04, d = 0.09 (Table 2)]. Furthermore, the trained limb circumferences significantly increased from the baseline to T1 [∆LC = +0.93 cm [0.63, 1.23], *p* = 0.01, d = 0.25 (Table 2)] and T2 (∆LC = +1.81 cm [1.55, 2.06], *p* = 0.001, d = 0.52 (Table 2)]. Additionally, the increase in circumference observed between T1 and T2 was statistically significant (∆LC = +0.88 cm [0.61, 1.14], *p* = 0.002, d = 0.24 (Table 2)]. Untrained limbs presented consistent subcutaneous skinfold thickness and limb circumferences across T0, T1, and T2 (*p* > 0.05). No significant differences were found in the control group for the abovementioned variables (*p* > 0.05).

**TABLE 2:** MVF, SST, and limb circumference.

Trained limb				
	T0	T1	T2	Significance
MVF (N)	244 ± 92	272 ± 99	290 ± 97	*#$
SST (mm)	5.2 ± 2.3	4.6 ± 1.9	4.4 ± 2.1	*#$
LC (cm)	27.5 ± 3.3	28.4 ± 3.8	29.3 ± 3.6	*#$

Untrained limb				
	T0	T1	T2	Significance
MVF (N)	260 ± 107	277 ± 112	286 ± 117	*#
SST (mm)	4.5 ± 2.3	4.7 ± 2.1	4.6 ± 2.0	none
LC (cm)	27.6 ± 3.7	27.7 ± 3.8	27.8 ± 3.7	none

CovF, coefficient of variation of force; LC, Limb circumference; MVF, maximal voluntary force; SST, subcutaneous skinfold thickness; T0, baseline; T1, after 4 weeks; T2, after 8 weeks. Within-group comparisons were performed with paired-sample t-tests. *, significant T0 - T1 result; #, significant T0 - T2 results; $, significant T1 - T2 results. Data are reported as the mean ± SD.

### 3.2 Motor unit identification

The number of the whole pool of identified motor units was 866 from all participants, 439 from the intervention and 427 from the control group, with no between-group differences at T0 (*p* = 0.627), T1 (*p* = 0.328), and T2 (*p* = 0.893). In addition, no within-group differences were observed in the number of identified motor units in the intervention (T0 - T1, *p* = 0.923; T0 - T2, *p* = 0.572; T1 - T2, *p* = 0.968) and control (T0 - T1, *p* = 0.627; T0 - T2, *p* = 0.157; T1 - T2, *p* = 0.150) groups. Of these, 103 (∼12%) were tracked across sessions, 56 motor units for the intervention and 47 for the control group. Per participant, 6.2 ± 1.78 motor units were tracked for the intervention and 5.2 ± 1.56 for the control group, with no between-group differences (*p* = 0.225). The number of identified and tracked motor units, classified by group, limb, and timeline, is reported in Table 3.

**TABLE 3:** Number of identified and tracked motor units.

	35%MVF			70%MVF			Total	Tracked
	T0	T1	T2	T0	T1	T2		
INT-TL	50	47	46	33	31	27	234	32 (13%)
INT-UL	42	45	44	30	29	28	218	24 (11%)
CNT-ND	46	42	43	29	28	28	216	28 (13%)
CNT-D	41	39	33	28	30	27	198	19 (10%)
Total	178	171	167	121	116	113	866	103 (12%)

CNT, control group; INT, intervention group; D, dominant limb; ND, non-dominant limb; T0, baseline; T1, after 4 weeks; T2, after 8 weeks; TL, trained limb; UL, untrained limb.

Compared to previous results investigating longitudinally tracked motor units following resistance training (Del Vecchio et al., 2019a), a lower percentage of tracked motor units was found due to more testing sessions (T0, T1, T2). This implicates a decrease in the number of possible motor units that can be tracked from the whole motor unit sample.

### 3.3 Motor unit adaptations

Changes in motor unit recruitment and derecruitment thresholds are reported in Table 4, whereas discharge rate comparisons are reported in Table 5. All the reported data refer to longitudinally tracked motor units as these reflect reliable intervention effects, whereas results from the whole sample may depend on the number of identified motor units (i.e., distribution) expressing specific properties following the decomposition process (Maathuis et al., 2008; Martinez-Valdes et al., 2017; Goodlich et al., 2023).

**TABLE 4:** Recruitment and derecruitment threshold adaptations.

Trained limbs				
	T0	T1	T2	Significance
RT (N)	99 ± 49	90 ± 40	83 ± 40	*#$
DT (N)	92 ± 52	86 ± 40	80 ± 38	*#$
RTr (%)	40 ± 12	34 ± 10	27 ± 8	*#$
DTr (%)	37 ± 13	32 ± 10	26 ± 7	*#$
Untrained limbs				
RT (N)	101 ± 54	95 ± 54	91 ± 49	*#
DT (N)	91 ± 55	83 ± 50	82 ± 45	*#
RTr (%)	39 ± 12	33 ± 11	32 ± 12	*#
DTr (%)	35 ± 13	30 ± 12	29 ± 13	*#

DT, derecruitment threshold; DTr, relative derecruitment threshold; RT, recruitment threshold; RTr, relative recruitment threshold; T0, baseline; T1, after 4 weeks; T2, after 8 weeks. Within-group comparisons were performed with mixed-effect linear regression model. *, significant T0 - T1 result; #, significant T0 - T2 results; $, significant T1 - T2 results. Data are reported as the mean ± SD.

**TABLE 5:** Discharge rate adaptations.

Trained limbs				
	T0	T1	T2	Significance
DR_MEAN_ (pps)	16.7 ± 4.5	20.2 ± 4.0	22.6 ± 4.3	*#$
DR_R_ (pps)	13.4 ± 3.6	12.9 ± 2.9	12.9 ± 3.2	none
DR_P_ (pps)	18.2 ± 4.4	20.8 ± 4.3	23.0 ± 3.3	*#$
DR_D_ (pps)	10.8 ± 2.8	11.1 ± 3.2	11.1 ± 2.8	none
Net-DR (pps)	4.8 ± 3.2	7.9 ± 4.8	10.1 ± 5.8	*#$
Untrained limbs				
DR_MEAN_ (pps)	20.6 ± 5.6	20.1 ± 4.2	20.6 ± 4.1	none
DR_R_ (pps)	16.7 ± 4.6	13.6 ± 4.7	13.3 ± 4.1	*#
DR_P_ (pps)	21.4 ± 5.2	21.4 ± 4.7	21.8 ± 4.3	none
DR_D_ (pps)	14.5 ± 3.1	11.5 ± 3.6	11.9 ± 0.7	*#
Net-DR (pps)	4.7 ± 3.4	7.8 ± 4.9	8.5 ± 5.3	*#

DR_D_, discharge rate at derecruitment; DR_MEAN_, mean discharge rate; DR_P_, discharge rate at plateau; DR_R_, discharge rate at recruitment; Net-DR, net discharge rate; T0, baseline; T1, after 4 weeks; T2, after 8 weeks. Within-group comparisons were performed with mixed-effect linear regression model. *, significant T0 - T1 result; #, significant T0 - T2 results; $, significant T1 - T2 results. Data are reported as the mean ± SD.

A significant group × limb × time interaction was observed for the absolute motor unit recruitment threshold (F_2, 281.34_ = 5.13, *p* = 0.003, pη^2^ = 0.15) and derecruitment threshold (F_2, 281.22_ = 4.42, *p* = 0.01, pη^2^ = 0.10). *Post hoc* analysis indicated a significant decline in recruitment threshold for trained limbs following the first 4 weeks [∆RT = −9 N [-13, −4], *p* < 0.001 (Table 4)], after 8 weeks [∆RT = −16 N [-20, −11], *p* < 0.001 (Table 4)], and between the fourth and eighth weeks [∆RT = −7 N [-9, −5], *p* = 0.001 (Table 4)]. Untrained limbs also presented a decrease in recruitment threshold after the first 4 weeks (∆RT = −6 N [-10, −1], *p* = 0.002 (Table 4)] and 8 weeks (∆RT = −10 [-14, −5], *p* < 0.001 (Table 4)], but no significant changes were observed between the fourth and eighth weeks (*p* = 0.115). Similarly, the absolute derecruitment threshold for trained limbs decreased significantly following the first 4 weeks (∆DT = −6 N [-11, −1], *p* = 0.01 (Table 4)], after 8 weeks (∆DT = −12 N [-17, −6], *p* < 0.001 (Table 4)], and between the fourth and the 8 weeks (∆DT = −6 N [-8, −3], *p* = 0.001 (Table 4)]. Untrained limbs exhibited a reduced derecruitment threshold after the first 4 weeks (∆DT = −8 N [-12, - 3], *p* = 0.01 (Table 4)] and 8 weeks (∆DT = −9 N [-14, −3], *p* = 0.004 (Table 4)], but no significant differences were found between the two timelines (*p* > 0.05).

We observed a significant group x limb × time interaction for the relative motor unit recruitment threshold (F_2, 274.35_ = 4.46, *p* = 0.01, pη^2^ = 0.11) and relative derecruitment threshold (F_2, 281.34_ = 3.93, *p* = 0.02, pη^2^ = 0.09). *Post hoc* results revealed a significant reduction in the relative recruitment threshold for trained limbs after the first 4 weeks [∆RTr = −6% [-7, −5], *p* < 0.001 (Table 4)], after 8 weeks (∆RTr = −12% [-14, −11], *p* < 0.001, Table 4), and between the fourth and the eighth weeks [∆RTr = −6% [-7, −5], *p* < 0.001 (Table 4)]. Untrained limbs also presented a reduced relative recruitment threshold following the first 4 weeks [∆RTr = −5% [-6, −4], *p* < 0.001 (Table 4)] and 8 weeks [∆RTr = −6% [-7, −5], *p* < 0.001 (Table 4)], with no significant differences between the fourth and the eighth weeks (*p* > 0.05). *Post hoc* analysis for the relative motor unit derecruitment threshold indicated a significant decrease for trained limbs following 4 weeks (∆DTr = −5% (-6, −3], *p* < 0.001 (Table 4)], after 8 weeks [∆DTr = −11% [-13, −8], *p* < 0.001 (Table 4)], and between the four and the eighth weeks [∆DTr = −6% [-7, −5], *p* < 0.001 (Table 4)]. In addition, untrained limbs exhibited a significant decline in the relative derecruitment threshold after the first 4 weeks [∆DTr = −5% [-6, −4], *p* < 0.001, Table 4] and 8 weeks [∆DTr = −5% [-7, −4], *p* < 0.001 (Table 4)], with no significant differences between the two timelines (*p* > 0.05).

A significant group x limb × time interaction was identified for the motor unit DR_MEAN_ (F_2, 281.93_ = 3.39, *p* = 0.03, pη^2^ = 0.08). *Post hoc* analysis indicated a significant increase exclusively in trained limbs after the first 4 weeks [∆DR = +3.4 pps [2.3, 4.6], *p* = 0.003 (Table 5)], 8 weeks [∆DR = +5.8 pps [4.6, 7.0], *p* < 0.001 (Table 5)], and between the two timelines [∆DR = +2.4 pps [1.3, 3.5], *p* = 0.01 (Table 5)]. A significant group x limb × time interaction was also observed in motor unit Net-DR (F_2, 282.77_ = 8.21, *p* < 0.001, pη^2^ = 0.19). *Post hoc* analysis revealed a significant increase in Net-DR for trained limbs following 4 weeks [∆DR = +3.1 pps (2.5, 3.7), *p* = 0.01 (Table 5)], 8 weeks [∆DR = +5.3 pps [4.3, 6.3), *p* < 0.001 (Table 5)], and between the fourth and the eighth weeks [∆DR = +2.2 pps (1.7, 2.7), *p* = 0.01 (Table 5)]. Untrained limbs also presented a significant increase in the Net-DR following the first 4 weeks [∆DR = +3.1 pps (2.5, 3.8), *p* = 0.01 (Table 5)] and 8 weeks [∆DR = +3.8 pps (2.9, 4.7), *p* < 0.001 (Table 5)], with no differences between these two timelines (*p* = 0.352).

We observed a significant group x limb x interaction for DR_R_ (F_2, 282.36_ = 4.32, *p* = 0.01, pη^2^ = 0.08), DR_P_ (F_2, 281.78_ = 10.27, *p* = 0.002, pη^2^ = 0.13), and DR_D_ (F_2, 279.15_ = 5.94, *p* = 0.003, pη^2^ = 0.10). *Post hoc* comparisons indicated a significant decrease in motor unit DR_R_ exclusively for untrained limbs following 4 weeks (∆DR = −3.1 pps (-4.5, −1.7), *p* = 0.02 (Table 5)) and 8 weeks (∆DR = −3.4 pps (-4.7, −2.0), *p* = 0.01 (Table 5)), with no differences between the two timelines (*p* = 0.403). *Post hoc* analysis for motor unit DR_P_ showed a significant increase only for trained limbs following 4 weeks (∆DR = +2.6 pps (1.4, 3.8), *p* = 0.02 (Table 5)), 8 weeks (∆DR = +4.1 pps (2.5, 3.8), *p* < 0.001 (Table 5)), and between week 4 and 8 (∆DR = +2.2 pps (1.1, 3.3), *p* = 0.01 (Table 5)). *Post hoc* comparisons indicated a significant decrease in motor unit DR_D_ only for untrained limbs following 4 weeks (∆DR = −3.0 pps (-4.0, −1.9), *p* = 0.02 (Table 5)) and 8 weeks (∆DR = −2.5 pps (-3.6, −1.5), *p* = 0.008 (Table 5)), with no differences between the two timelines (*p* = 0.365).

A group × time interaction was found for ISIv (F_2, 280.57_ = 11.76, *p* < 0.001, pη^2^ = 0.16). *Post hoc* analyses revealed a significant decrease in ISIv for trained limbs after 4 weeks (∆ISIv = −6.5% (-7.8, −5.2), *p* = 0.001), after 8 weeks (∆ISIv = −9.5% (-10.8, −8.3), *p* < 0.001), and between the fourth and the eighth weeks (∆ISIv = −3.0% (-4.2, −1.9), *p* = 0.001). Furthermore, the untrained limb presented a significant decrease in ISIv following 4 weeks (∆ISIv = −3.9% (-5.4, −2.4), *p* = 0.004) and 8 weeks (∆ISIv = −4.6% (-6.2, −3.91, *p* < 0.001), with no differences between the fourth and the eighth weeks (*p* = 0.401).

No changes were observed in the control group for motor unit properties (*p* > 0.05).

### 3.4 Neuromechanical associations

**FIGURE 3:**
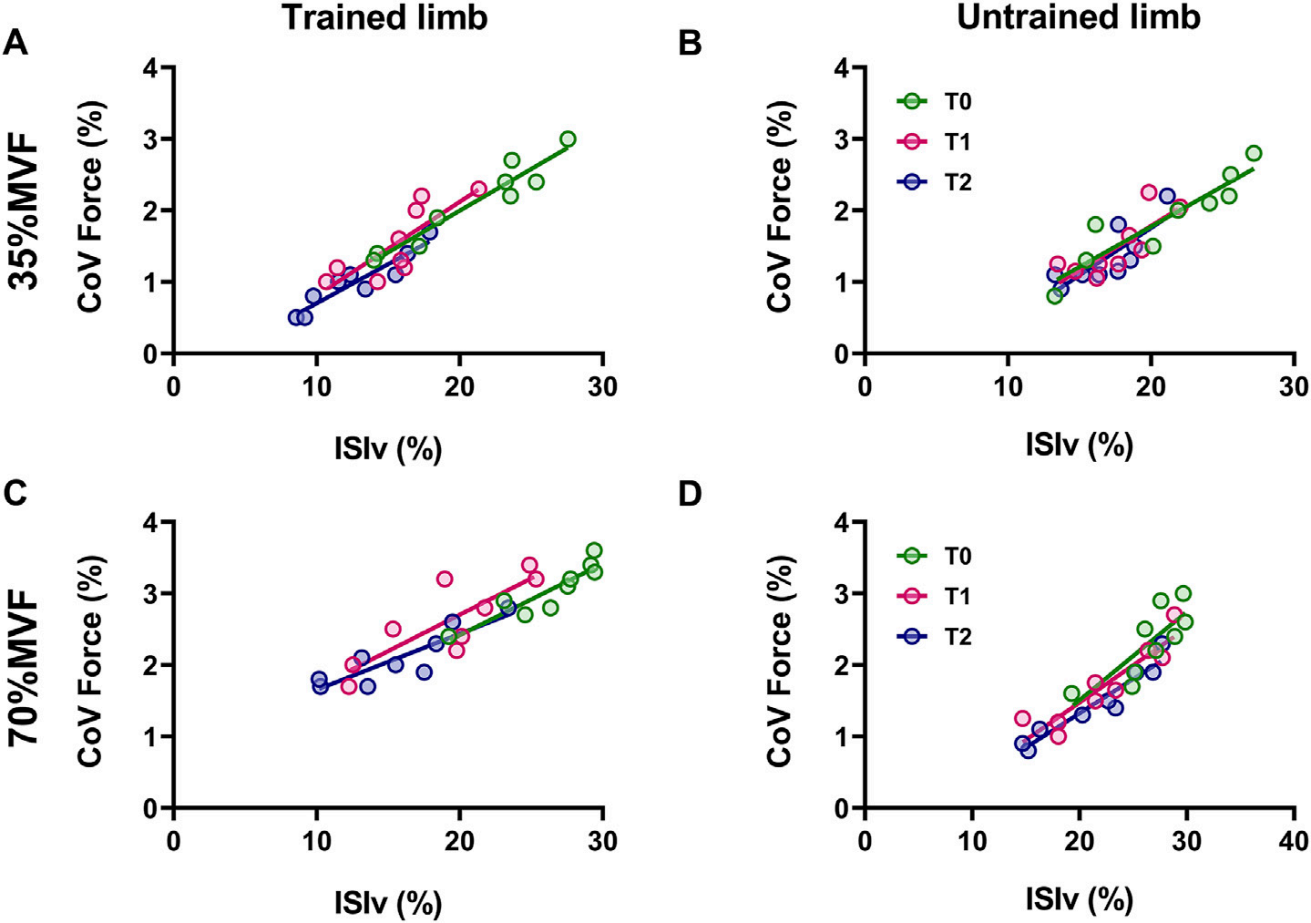
The association between the mean interspike interval variability and coefficient of variation of force Scatter plots of the relationship between the ISIv and CovF are displayed for trained and untrained limbs at 35% MVF **(A, B)** and 70% MVF **(C, D)**. Each marker represents a single participant value, reflecting the average ISIv for motor units and CovF at T0, T1, and T2. Tracking procedures ensure the reliability of comparisons.

The relationship between interspike interval variability and the coefficient of variation of force (i.e., firing and force fluctuation) was statistically significant across all time points and intensities for both limbs (Figure 3). At 35% MVF, trained limbs demonstrated a strong to very strong coefficient of determination at T0 (*R* ^2^ = 0.92, *p* < 0.001), T1 (*R* ^2^ = 0.66, *p* = 0.007), and T2 (*R* ^2^ = 0.87, *p* < 0.001), with no significant differences in slopes (*p* = 0.818). Similarly, untrained limbs exhibited strong to very strong association at T0 (*R* ^2^ = 0.85, *p* < 0.001), T1 (*R* ^2^ = 0.69, *p* = 0.01), and T2 (*R* ^2^ = 0.79, *p* = 0.007), with consistent slopes across time points (*p* = 0.875). At 70% MVF, trained limbs presented a strong to very strong coefficient of determination at T0 (*R* ^2^ = 0.82, *p* < 0.001), T1 (*R* ^2^ = 0.68, *p* = 0.005), and T2 (*R* ^2^ = 0.77, *p* = 0.001), with no differences in slopes (*p* = 0.673). Untrained limbs also exhibited a strong to very strong coefficient of determination at T0 (*R* ^2^ = 0.64, *p* = 0.008), T1 (*R* ^2^ = 0.84, *p* < 0.001), and T2 (*R* ^2^ = 0.91, *p* < 0.001), with consistent slopes across the timelines (*p* = 0.681).

**FIGURE 4:**
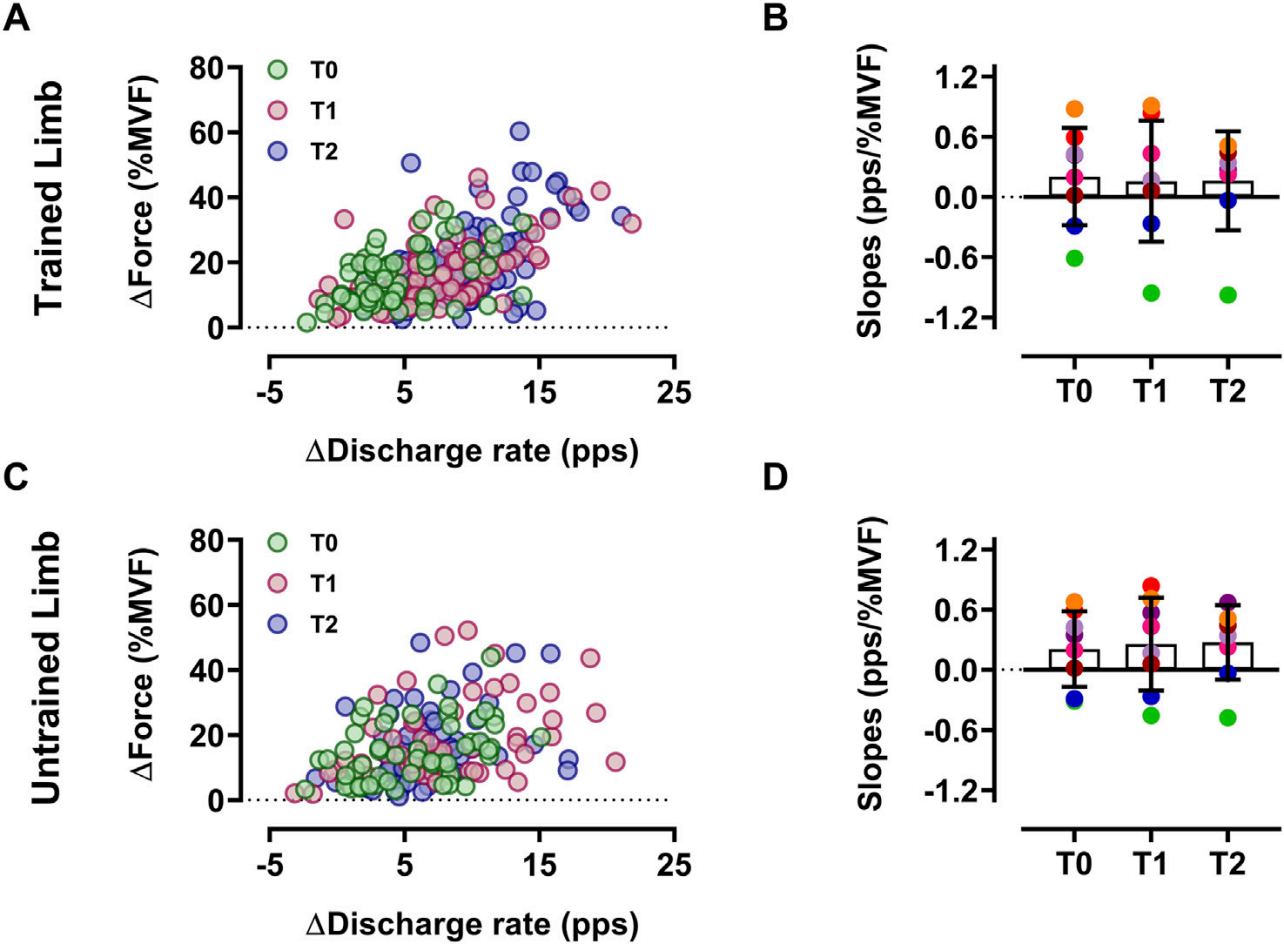
Input-output gain of motoneurons Scatter plot of the input-output gain of the ∆-DR (plateau *minus* recruitment) as a function of the ∆-Force (plateau force *minus* recruitment threshold force) reflecting the input-output gain of motoneurons of trained **(A)** and untrained **(C)** limbs. Each circle represents a motor unit, and each color refers to a timeline. Slope comparisons between T0, T1, and T2 are displayed for each participant’s trained **(B)** and untrained **(D)** limbs. Each colored marker represents a single participant’s slope value. Data are reported as the mean ± SD.

**FIGURE 5:**
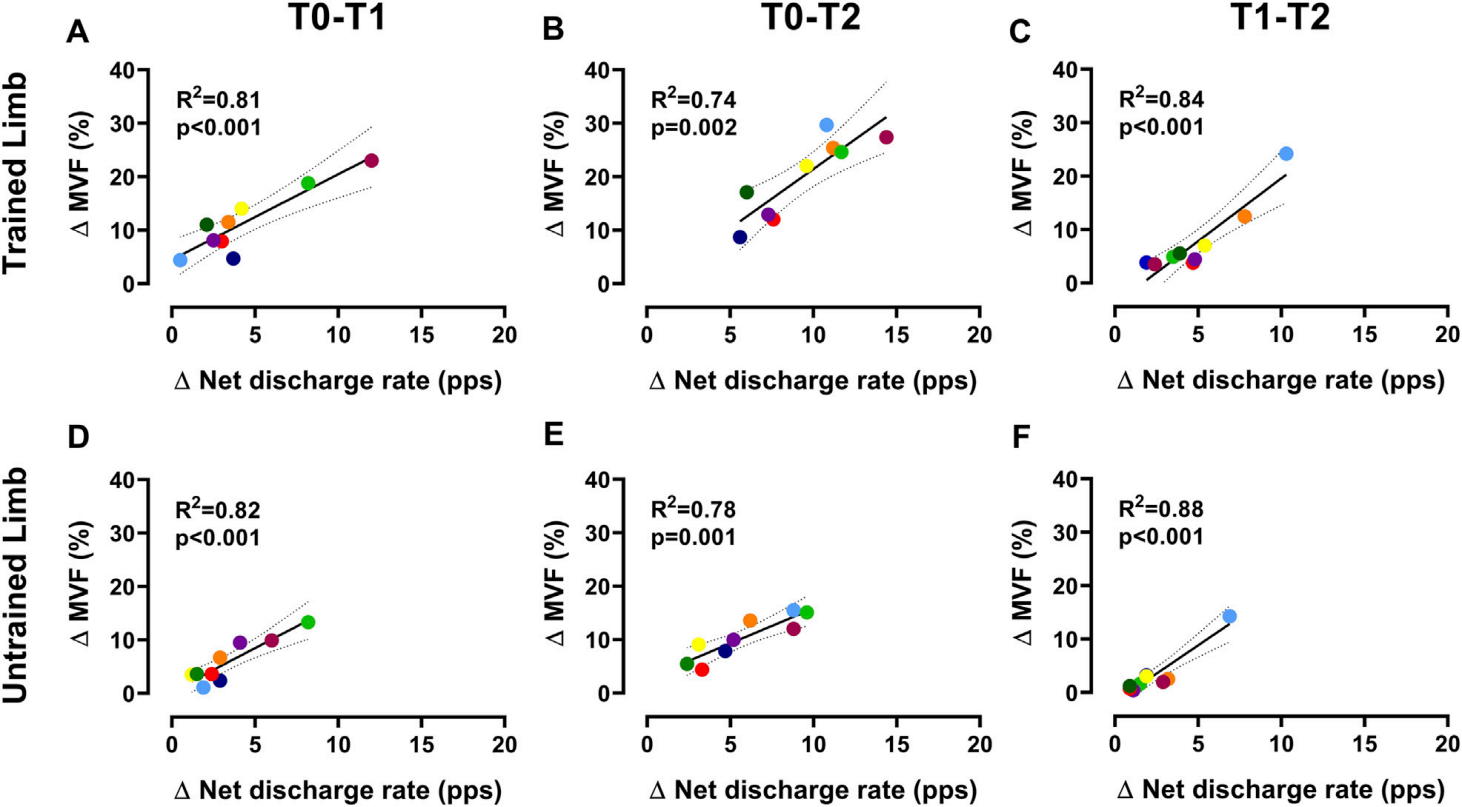
Association between ∆net discharge rate and ∆MVF Scatter plots displaying the association between the change in the *net discharge rate* of motor units (i.e., the gain in the discharge rate from recruitment to target force) and MVF for trained **(A–C)** and untrained **(D–F)** sides. Each marker represents a single participant value, reflecting the average change in the *net discharge rate* of motor units and MVF for all regressions. Tracking procedures ensure the reliability of comparisons by averaging the net discharge rate of the same motor units.

The input-output gain of motoneurons, measured as the association between the change in discharge rate and exerted force during the ramp-up phase of contractions, was not altered by resistance training in both limbs for the intervention group (*p* > 0.05 (Figure 4)). Similarly, the control group showed no significant differences (*p* > 0.05). In trained limbs, we found a strong linear association between the change (i.e., delta) in the *net discharge rate* and the maximal voluntary force between T0 and T1 (*R* ^2^ = 0.81, *p* < 0.001 (Figure 5A)), T0 and T2 (*R* ^2^ = 0.74, *p* = 0.002 (Figure 5B)), as well as between T1 and T2 (*R* ^2^ = 0.84, *p* < 0.001 (Figure 5C)). We also found a strong linear association between the change in the net discharge rate and the maximal voluntary force in the untrained side between T0 and T1 (*R* ^2^ = 0.82, *p* < 0.001 (Figure 5D)), T0 and T2 (*R* ^2^ = 0.78, *p* = 0.001 (Figure 5E)), as well as between T1 and T2 (*R* ^2^ = 0.72, *p* = 0.003 (Figure 5F)).

## 4 Discussion

The main finding of this study is that strength increases in the untrained muscles were accompanied by specific adaptations in motor unit properties. Notably, we observed an increase in the *net discharge rate* in the untrained limb, reflecting a greater net motoneuron output to the muscle. This parameter is particularly relevant as it represents the gain in discharge rate from when a motor unit is activated to reaching the target force, an adaptation also occurring on the trained side. Higher muscle strength was also accompanied by a lower recruitment threshold and reduced interspike interval variability, indicative of an increased cumulative motor unit contribution to force exertion and enhanced regularity in the discharge activity in both limbs. Furthermore, these modifications were observed in longitudinally tracked motor units, which ensures the robustness of our findings by offering a unique perspective of neuromuscular adaptations underlying cross-education.

The number of longitudinally tracked motor units was lower than in previous studies investigating resistance training adaptations (Del Vecchio et al., 2019a). This discrepancy is primarily attributed to the analyzed muscle (*biceps-brachii* in the present study) and the number of tracking sessions (*three timelines*), both of which have been reported to influence the number of identified and tracked motor units (Del Vecchio et al., 2020). Indeed, the decomposition of HDsEMG signals from biceps brachii typically implicates lower identified motor units compared to other muscles (e.g., *tibialis anterior*), and the likelihood to reidentify the same motor units decreases with the increase in the number of sessions.

The force output of dominant and non-dominant biceps brachii was consistent with values expected from recreationally active individuals (Casolo et al., 2021). The significant strength gains observed in trained muscles after four and 8 weeks of intervention, 11% at T1 and 19% at T2, aligned with established neuromechanical adaptations associated with resistance training (Folland and Williams, 2007) and were accompanied by significant increases in limb circumference across testing sessions. Untrained limbs also presented a noteworthy enhancement in MVF with no changes in limb circumference, comparable to that observed in previous investigations exploring cross-education in the biceps brachii (Green and Gabriel, 2018; Manca et al., 2018). These findings support prior research indicating that initial strength increases are predominantly driven by neural adaptations (Folland and Williams, 2007; Balshaw et al., 2017), underscoring the significant role of eccentric contraction in eliciting cross-education effects (Altheyab et al., 2024). Nevertheless, similar force output between T1 and T2 in untrained limbs suggests that primary adaptations supporting cross-education occur in the first 4 weeks of resistance training.

The observed strength enhancements were accompanied by various motor unit adaptations. First, the recruitment threshold was significantly lower in trained limbs, resulting in an earlier activation of the motor unit pool, which aligned with prior findings (Del Vecchio et al., 2019a). Similar effects were observed in the contralateral homologous muscle, supporting the hypotheses regarding the influences from contralateral networks affecting neural activity on the untrained side (Lee and Carroll, 2007; Leung et al., 2018). Furthermore, motor unit recruitment threshold is known to depend on the relative share of the synaptic input to motoneurons and their intrinsic threshold properties (Heckman et al., 2009). It has been suggested that changes in the distribution of excitation levels activating motor units are often a result of neural adaptations to resistance training (Dideriksen and Del Vecchio, 2023), leading to a lower excitatory input required for motor unit activation. Since cross-education has been associated with gain transfer occurring at spinal and supraspinal levels, it is plausible that lower recruitment thresholds observed in both limbs stem from either or both enhanced synaptic input and intrinsic threshold properties (Heckman et al., 2009; Del Vecchio et al., 2019a; Škarabot et al., 2021; Dideriksen and Del Vecchio, 2023). In addition, lower derecruitment thresholds were observed, likely depending on the type of resistance training and, similarly to recruitment, the intrinsic threshold properties of motor units (Heckman et al., 2009). Our results indicate that chronic eccentric-contraction training significantly decreased recruitment and derecruitment thresholds, supporting previous findings investigating the effects of acute eccentric contractions on motor unit properties (Dartnall et al., 2009; Hayman et al., 2024).

The lower ISIv accompanied by increased strength in both limbs potentially depends on the hormonal response typically observed following strength training (Handelsman et al., 2018). This adaptation is associated with reduced potential dispersion during firing activity (Guo et al., 2022), resulting in a decreased firing fluctuation and aligning with previous evidence indicating reduced firing variability in response to resistance training (Vila-Chã and Falla, 2016). Lower interspike interval variability was also accompanied by enhanced force steadiness (*lower CovF*), supporting findings involving the strong association between these two variables (Dideriksen et al., 2012; Enoka and Farina, 2021). Indeed, both limbs exhibited strong ISIv-CovF relationships, which were not altered by resistance training, highlighting that gain transfer regards not only muscle strength but also force steadiness.

The higher discharge rate observed during the plateau phase of contractions in trained limbs likely stems from the enhanced neural drive required to generate higher absolute forces, as suggested in previous studies (Del Vecchio et al., 2019a). In contrast, we found a consistent discharge rate at the plateau and a lower discharge rate at recruitment in untrained limbs. It is plausible that these divergent responses depend on a differential modification in musculotendinous stiffness, which has been demonstrated to decrease in trained muscles (Grosset et al., 2009). This reduction affects neuromechanical delay and motor unit discharge rates at the activation by altering the stretch response of the muscle-tendon unit as muscle fibers depolarize (Del Vecchio et al., 2018; Del Vecchio et al., 2019a). Indeed, lower musculotendinous stiffness is associated with a higher discharge rate at recruitment to compensate for the slack in passive component response (Stein and Parmiggiani, 1979; Linden et al., 1991; Pasquet et al., 2005). Considering lower recruitment thresholds, it is plausible that motoneuron starts sending action potentials at a lower excitatory input in both limbs (Del Vecchio et al., 2019a; Dideriksen and Del Vecchio, 2023). This suggests that the discharge rate at recruitment may be lower in conditions of consistent muscle stiffness. Instead, trained muscles will maintain a similar discharge rate at recruitment with decreased stiffness. This also aligns with the hypothesis that resistance training elicits increased *rate coding gain* (i.e., the sensitivity of motoneurons to increase their spike frequency from recruitment to the plateau), resulting in a steeper rise in discharge rate once motoneurons begin firing, explaining the gain in the discharge rate from the activation to plateau (i.e., *net discharge rate*) achieved through differential discharge adaptations on both trained and untrained sides (Dideriksen and Del Vecchio, 2023).

The input-output gain of motoneurons was not altered by training, a finding aligning with previous evidence (Nuzzo et al., 2017; Del Vecchio et al., 2019a). The similar association between the change in discharge rate and exerted force implies a similar motoneuron output to a neural input following the intervention (Devanne et al., 1997). This suggests that the observed discharge adaptations may occur to reach a greater absolute force, underlining that mechanical modulations of both trained and untrained limbs are associated with corresponding changes in motor unit discharge characteristics. Indeed, both sides presented a higher *net discharge rate*, which is the gain in the discharge rate from when the motoneuron starts firing to the value of the target force. Furthermore, the strong association between the change in the *net discharge rate* and MVF highlights that the increase in muscle strength is strongly related to this parameter in both limbs. These findings support the evidence that greater muscle force is strongly associated with higher net spinal motoneuron output to muscles (Del Vecchio et al., 2019a; Nuccio et al., 2021).

In summary, our results indicate that unilateral resistance training of the biceps brachii results in significant neuromechanical adaptations, evidenced by higher maximal voluntary force and changes in motor unit characteristics in both trained and untrained limbs. The main findings include an increase in *net discharge rate*, lower recruitment thresholds, and reduced interspike interval variability, all indicative of neural adaptations occurring in both limbs, with untrained limbs presenting enhancements in the first 4 weeks of unilateral resistance training. The strong association between the increase in maximal strength and the net spinal motoneuron output to muscles underscores that changes in motor unit discharge characteristics potentially mediate the observed gains. These results also underline the role of neural adaptations in early strength improvements, potentially contributing to the cross-education phenomenon.

## 5 Limitations and future studies

Although the differential adaptations of motor units tracked before and after training have been characterized in the present study, it was not possible to directly assess spinal and central modulation to determine these changes, representing the main limitation in identifying multiple possible sites of adaptations. Another limitation is that cross-education was explored over a relatively brief period. To address this, future studies could include more time points within the first 4 weeks to better assess the kinetics of neural modifications. Future investigations involving the concurrent measurement of brain activity and spinal output may provide novel insights into the neural adaptations to unilateral strength training. Additionally, exploring the long-term sustainability of cross-education effects and their applicability to diverse populations will contribute to a more comprehensive understanding of this phenomenon.

## Data Availability

The raw data supporting the conclusions of this article will be made available by the authors, without undue reservation.

## References

[B1] Altheyab A. Alqurashi H. England T. J. Phillips B. E. Piasecki M. (2024). Cross-education of lower limb muscle strength following resistance exercise training in males and females: a systematic review and meta-analysis. Exp. Physiol. 10.1113/EP091881 PMC13238690 39235953

[B2] Amstrong R. B. (1984). Mechanisms of exercise-induced delayed onset muscular soreness: a brief review. Med. Sci. Sports Exerc. 16 (6), 529–538. 10.1249/00005768-198412000-00002 6392811

[B3] Andrade J. M. Estévez-Pérez M. G. (2014). Statistical comparison of the slopes of two regression lines: a tutorial. Anal. Chim. Acta 838, 1–12. 10.1016/j.aca.2014.04.057 25064237

[B4] Ansdell P. Brownstein C. G. Škarabot J. Angius L. Kidgell D. Frazer A. (2020). Task-specific strength increases after lower-limb compound resistance training occurred in the absence of corticospinal changes in vastus lateralis. Exp. Physiol. 105 (7), 1132–1150. 10.1113/EP088629 32363636

[B5] Balshaw T. G. Massey G. J. Maden-Wilkinson T. M. Morales-Artacho A. J. McKeown A. Appleby C. L. (2017). Changes in agonist neural drive, hypertrophy and pre-training strength all contribute to the individual strength gains after resistance training. Eur. J. Appl. Physiology 117 (4), 631–640. 10.1007/s00421-017-3560-x 28239775

[B6] Barss T. S. Pearcey G. E. P. Zehr P. E. (2016). Cross-education of strength and skill: an old idea with applications in the aging nervous system. yale J. Biol. Med. 89, 81–86. Available at: https://www.researchgate.net/publication/291969898. 27505019 PMC4797840

[B7] Bouguetoch A. Martin A. Grosprêtre S. (2021). Does partial activation of the neuromuscular system induce cross-education training effect? Case of a pilot study on motor imagery and neuromuscular electrical stimulation. Eur. J. Appl. Physiology 121 (8), 2337–2348. 10.1007/s00421-021-04710-8 33997913

[B8] Boyes N. G. Yee P. Lanovaz J. L. Farthing J. P. (2017). Cross-education after high-frequency versus low-frequency volume-matched handgrip training. Muscle Nerve 56 (4), 689–695. 10.1002/mus.25637 28249351

[B9] Bundy D. T. Leuthardt E. C. (2019). The cortical physiology of ipsilateral limb movements. Trends Neurosci. 42, 825–839. 10.1016/j.tins.2019.08.008 31514976 PMC6825896

[B10] Burrows M. Peters C. E. (2007). The influence of oral contraceptives on athletic performance in female athletes. Sports Med. 37, 557–574. 10.2165/00007256-200737070-00001 17595152

[B11] Carr J. C. Ye X. Stock M. S. Bemben M. G. DeFreitas J. M. (2019). The time course of cross-education during short-term isometric strength training. Eur. J. Appl. physiology 119, 1395–1407. 10.1007/s00421-019-04130-9 30949806

[B12] Carson R. G. (2005). Neural pathways mediating bilateral interactions between the upper limbs. Brain Res. Rev. 49, 641–662. 10.1016/j.brainresrev.2005.03.005 15904971

[B13] Casolo A. Del Vecchio A. Balshaw T. G. Maeo S. Lanza M. B. Felici F. (2021). Behavior of motor units during submaximal isometric contractions in chronically strength-trained individuals. J. Appl. Physiology 131, 1584–1598. 10.1152/japplphysiol.00192.2021 34617822

[B14] Correll J. Mellinger C. Pedersen E. J. (2022). Flexible approaches for estimating partial eta squared in mixed-effects models with crossed random factors. Behav. Res. Methods 54 (4), 1626–1642. 10.3758/s13428-021-01687-2 34561820

[B15] Dartnall T. J. Nordstrom M. A. Semmler J. G. (2008). Motor unit synchronization is increased in biceps brachii after exercise-induced damage to elbow flexor muscles. J. Neurophysiology 99 (2), 1008–1019. 10.1152/jn.00686.2007 18171708

[B16] Dartnall T. J. Rogasch N. C. Nordstrom M. A. Semmler J. G. (2009). Eccentric muscle damage has variable effects on motor unit recruitment thresholds and discharge patterns in elbow flexor muscles. J. Neurophysiology 102 (1), 413–423. 10.1152/jn.91285.2008 19420118

[B17] Del Vecchio A. Casolo A. Negro F. Scorcelletti M. Bazzucchi I. Enoka R. (2019a). The increase in muscle force after 4 weeks of strength training is mediated by adaptations in motor unit recruitment and rate coding. J. Physiol. 597 (7), 1873–1887. 10.1113/JP277250 30727028 PMC6441907

[B18] Del Vecchio A. Enoka R. M. Farina D. (2024) Specificity of early motor unit adaptations with resistive exercise training. J. Physiol. 0, 2679–2688. 10.1113/JP282560 38686581

[B19] Del Vecchio A. Holobar A. Falla D. Felici F. Enoka R. M. Farina D. (2020). Tutorial: analysis of motor unit discharge characteristics from high-density surface EMG signals. J. Electromyogr. Kinesiol. 53, 102426. 10.1016/j.jelekin.2020.102426 32438235

[B20] Del Vecchio A. Negro F. Holobar A. Casolo A. Folland J. P. Felici F. (2019b). You are as fast as your motor neurons: speed of recruitment and maximal discharge of motor neurons determine the maximal rate of force development in humans. J. Physiology 597 (9), 2445–2456. 10.1113/JP277396 PMC6487919 30768687

[B21] Del Vecchio A. Úbeda A. Sartori M. Azorín J. M. Felici F. Farina D. (2018). Central nervous system modulates the neuromechanical delay in a broad range for the control of muscle force. J. Appl. Physiology 125 (5), 1404–1410. 10.1152/japplphysiol.00135.2018 29975604

[B22] Devanne H. Lavoie B. Capaday C. (1997). Input-output properties and gain changes in the human corticospinal pathway. Exp. Brain Res. 114 (2), 329–338. 10.1007/pl00005641 9166922

[B23] Dideriksen J. Del Vecchio A. (2023). Adaptations in motor unit properties underlying changes in recruitment, rate coding, and maximum force. J. neurophysiology 129 (1), 235–246. 10.1152/jn.00222.2022 36515411

[B24] Dideriksen J. L. Negro F. Enoka R. M. Farina D. (2012). Motor unit recruitment strategies and muscle properties determine the influence of synaptic noise on force steadiness. J. Neurophysiology 107 (12), 3357–3369. 10.1152/jn.00938.2011 22423000 PMC3378401

[B25] Duchateau J. Semmler J. G. Enoka R. M. (2006). Training adaptations in the behavior of human motor units. J. Appl. Physiology 101, 1766–1775. 10.1152/japplphysiol.00543.2006 16794023

[B26] Elgueta-Cancino E. Evans E. Martinez-Valdes E. Falla D. (2022). The effect of resistance training on motor unit firing properties: a systematic review and meta-analysis. Front. Physiology 13, 817631. 10.3389/fphys.2022.817631 PMC8918924 35295567

[B27] Elliott-Sale K. J. McNulty K. L. Ansdell P. Goodall S. Hicks K. M. Thomas K. (2020). The effects of oral contraceptives on exercise performance in women: a systematic review and meta-analysis. Sports Med. 50 (10), 1785–1812. 10.1007/s40279-020-01317-5 32666247 PMC7497464

[B28] Enoka R. M. Farina D. (2021). Force steadiness: from motor units to voluntary actions. Physiology 36 (2), 114–130. 10.1152/physiol.00027.2020 33595382

[B29] Farthing J. P. Borowsky R. Chilibeck P. D. Binsted G. Sarty G. E. (2007). Neuro-physiological adaptations associated with cross-education of strength. Brain Topogr. 20 (2), 77–88. 10.1007/s10548-007-0033-2 17932739

[B30] Farthing J. P. Chilibeck P. D. (2003). The effect of eccentric training at different velocities on cross-education. Eur. J. Appl. Physiology 89 (6), 570–577. 10.1007/s00421-003-0841-3 12756570

[B31] Ferguson K. A. Cardin J. A. (2020). Mechanisms underlying gain modulation in the cortex. Nat. Rev. Neurosci. 21, 80–92. 10.1038/s41583-019-0253-y 31911627 PMC7408409

[B32] Fimland M. S. Helgerud J. Solstad G. M. Iversen V. M. Leivseth G. Hoff J. (2009). Neural adaptations underlying cross-education after unilateral strength training. Eur. J. Appl. Physiology 107 (6), 723–730. 10.1007/s00421-009-1190-7 19756705

[B33] Folland J. P. Williams A. G. (2007). The adaptations to strength training: morphological and neurological contributions to increased strength. Sports Med. 37, 145–168. 10.2165/00007256-200737020-00004 17241104

[B34] Frazer A. K. Pearce A. J. Howatson G. Thomas K. Goodall S. Kidgell D. J. (2018). Determining the potential sites of neural adaptation to cross-education: implications for the cross-education of muscle strength. Eur. J. Appl. Physiology 118, 1751–1772. 10.1007/s00421-018-3937-5 29995227

[B35] Goodlich B. I. Del Vecchio A. Kavanagh J. J. (2023). Motor unit tracking using blind source separation filters and waveform crosscorrelations: reliability under physiological and pharmacological conditions. J. Appl. Physiology 135 (2), 362–374. 10.1152/japplphysiol.00271.2023 37410901

[B36] Green L. A. Gabriel D. A. (2018). The cross education of strength and skill following unilateral strength training in the upper and lower limbs. J. Neurophysiol. 120, 468–479. 10.1152/jn.00116.2018 29668382 PMC6139459

[B37] Green L. A. Gabriel D. A. (2018). The effect of unilateral training on contralateral limb strength in young, older, and patient populations: a meta-analysis of cross education. Phys. Ther. Rev. 23 (4–5), 238–249. 10.1080/10833196.2018.1499272

[B38] Grosset J. F. Piscione J. Lambertz D. Pérot C. (2009). Paired changes in electromechanical delay and musculo-tendinous stiffness after endurance or plyometric training. Eur. J. Appl. Physiology 105 (1), 131–139. 10.1007/s00421-008-0882-8 18853177

[B39] Guo Y. Piasecki J. Swiecicka A. Ireland A. Phillips B. E. Atherton P. J. (2022). Circulating testosterone and dehydroepiandrosterone are associated with individual motor unit features in untrained and highly active older men. GeroScience 44 (3), 1215–1228. 10.1007/s11357-021-00482-3 34862585 PMC9213614

[B40] Handelsman D. J. Hirschberg A. L. Bermon S. (2018). “Circulating testosterone as the hormonal basis of sex differences in athletic performance, Endocrine Reviews,”. Oxford University Press, 803–829. 10.1210/er.2018-00020 PMC6391653 30010735

[B41] Harput G. Ulusoy B. Yildiz T. I. Demirci S. Eraslan L. Turhan E. (2018). Cross-education improves quadriceps strength recovery after ACL reconstruction: a randomized controlled trial. Knee Surg. Sports Traumatol. Arthrosc. 27 (1), 68–75. 10.1007/s00167-018-5040-1 29959448

[B42] Hayman O. Ansdell P. Angius L. Thomas K. Horsbrough L. Howatson G. (2024). Changes in motor unit behaviour across repeated bouts of eccentric exercise. Exp. Physiol. Prepr. 109, 1896–1908. 10.1113/EP092070 PMC11522828 39226215

[B43] Heckman C. J. Mottram C. Quinlan K. Theiss R. Schuster J. (2009). Motoneuron excitability: the importance of neuromodulatory inputs. Clin. Neurophysiol. 120, 2040–2054. 10.1016/j.clinph.2009.08.009 19783207 PMC7312725

[B44] Hendy A. M. Lamon S. (2017). The cross-education phenomenon: brain and beyond. Front. Physiology. Front. Res. Found. 8, 297. 10.3389/fphys.2017.00297 PMC5423908 28539892

[B45] Holobar A. Farina D. (2014). Blind source identification from the multichannel surface electromyogram. Physiol. Meas. 35, 143–165. 10.1088/0967-3334/35/7/R143 24943407

[B46] Holobar A. Farina D. Gazzoni M. Merletti R. Zazula D. (2014). Estimating motor unit discharge patterns from high-density surface electromyogram. Clin. Neurophysiol. 120 (3), 551–562. 10.1016/j.clinph.2008.10.160 19208498

[B47] Holobar A. Zazula D. (2007). Multichannel blind source separation using convolution kernel compensation. IEEE Trans. signal Process. 55 (9), 4487–4496. 10.1109/tsp.2007.896108

[B48] Hortobágyi T. Richardson S. P. Lomarev M. Shamim E. Meunier S. Russman H. (2011). Interhemispheric plasticity in humans. Med. Sci. Sports Exerc. 43 (7), 1188–1199. 10.1249/MSS.0b013e31820a94b8 21200340 PMC4137570

[B49] Howatson G. Taylor M. B. Rider P. Motawar B. R. McNally M. P. Solnik S. (2011). Ipsilateral motor cortical responses to TMS during lengthening and shortening of the contralateral wrist flexors. Eur. J. Neurosci. 33 (5), 978–990. 10.1111/j.1460-9568.2010.07567.x 21219480 PMC3075420

[B50] Huang C. Klein C. S. Meng Z. Zhang Y. Li S. Zhou P. (2019). Innervation zone distribution of the biceps brachii muscle examined using voluntary and electrically-evoked high-density surface EMG. J. NeuroEngineering Rehabilitation 16 (1), 73. 10.1186/s12984-019-0544-6 PMC6560814 31186009

[B51] Hug F. Avrillon S. Del Vecchio A. Casolo A. Ibanez J. Nuccio S. (2021). Analysis of motor unit spike trains estimated from high-density surface electromyography is highly reliable across operators. J. Electromyogr. Kinesiol. 58, 102548. 10.1016/j.jelekin.2021.102548 33838590

[B52] Kang M. Ragan B. G. Park J. H. (2008). Issues in outcomes research: an overview of randomization techniques for clinical trials. J. Athl. Train. 43, 215–221. 10.4085/1062-6050-43.2.215 18345348 PMC2267325

[B53] Kay A. D. Blazevich A. J. Tysoe J. C. Baxter B. A. (2024). Cross-education effects of isokinetic eccentric plantarflexor training on flexibility, strength, and muscle-tendon mechanics. Med. Sci. Sports Exerc. 56 (7), 1242–1255. 10.1249/MSS.0000000000003418 38451696

[B54] Kramer W. J. Ratamess N. A. French D. N. (2002). Resistance training for health and performance. Curr. sports Med. Rep. 1 (3), 165–171. 10.1249/00149619-200206000-00007 12831709

[B55] Latella C. Kidgell D. J. Pearce A. J. (2012). Reduction in corticospinal inhibition in the trained and untrained limb following unilateral leg strength training. Eur. J. Appl. Physiol. 112, 3097–3107. 10.1007/s00421-011-2289-1 22200796

[B56] Lecce E. Conti A. Nuccio S. Felici F. Bazzucchi I. (2024a). Characterising sex‐related differences in lower‐ and higher‐threshold motor unit behaviour through high‐density surface electromyography. Exp. Physiol. 109 (8), 1317–1329. 10.1113/EP091823 38888901 PMC11291872

[B57] Lecce E. Del Vecchio A. Francesco Felici S. N. Bazzucchi I. (2024). Higher dominant muscle strength is mediated by motor unit discharge rates and proportion of common synaptic inputs. PREPRINT (Version 1). 10.21203/rs.3.rs-5317484/v1 PMC11894131 40065078

[B58] Lecce E. Nuccio S. Del Vecchio A. Conti A. Nicolò A. Sacchetti M. (2023a). Sensorimotor integration is affected by acute whole-body vibration: a coherence study. Front. Physiology 14, 1266085. 10.3389/fphys.2023.1266085 PMC10523146 37772061

[B59] Lecce E. Nuccio S. Del Vecchio A. Conti A. Nicolò A. Sacchetti M. (2023b). The acute effects of whole-body vibration on motor unit recruitment and discharge properties. Front. Physiology 14, 1124242. 10.3389/fphys.2023.1124242 PMC9988902 36895636

[B60] Lecce E. Romagnoli R. Frinolli G. Felici F. Piacentini M. F. Bazzucchi I. (2024b). Exerting force at the maximal speed drives the increase in power output in elite athletes after 4 weeks of resistance training. Eur. J. Appl. Physiology Prepr. 10.1007/s00421-024-05604-1 39266729

[B61] Lee M. Carroll T. J. (2007). Cross education possible mechanisms for the contralateral effects of unilateral resistance training. Sports Med. 37, 1–14. 10.2165/00007256-200737010-00001 17190532

[B62] Leung M. Rantalainen T. Teo W. P. Kidgell D. (2018). The ipsilateral corticospinal responses to cross-education are dependent upon the motor-training intervention. Exp. Brain Res. 236 (5), 1331–1346. 10.1007/s00221-018-5224-4 29511785

[B63] Linden D. W. V. Kukulka C. G. Soderberg G. L. (1991). The effect of muscle length on motor unit discharge characteristics in human tibialis anterior muscle. Exp. Brain Res. 84, 210–218. 10.1007/BF00231776 1855559

[B64] Maathuis E. M. Drenthen J. van Dijk J. P. Visser G. H. Blok J. H. (2008). Motor unit tracking with high-density surface EMG. J. Electromyogr. Kinesiol. 18 (6), 920–930. 10.1016/j.jelekin.2008.09.001 18996724

[B65] Manca A. Hortobágyi T. Carroll T. J. Enoka R. M. Farthing J. P. Gandevia S. C. (2021). Contralateral effects of unilateral strength and skill training: modified delphi consensus to establish key aspects of cross-education. Sports Med. 51 (1), 11–20. 10.1007/s40279-020-01377-7 33175329 PMC7806569

[B66] Manca A. Hortobágyi T. Rothwell J. Deriu F. (2018). Neurophysiological adaptations in the untrained side in conjunction with cross-education of muscle strength: a systematic review and meta-analysis. J. Appl. Physiology 124, 1502–1518. 10.1152/japplphysiol.01016.2017 29446711

[B67] Martinez F. Abián P. Jiménez F. Abián-Vicén J. (2021). Effects of cross-education after 6 Weeks of eccentric single-leg decline squats performed with different execution times: a randomized controlled trial. Sports Health 13 (6), 594–605. 10.1177/19417381211016353 34075821 PMC8559000

[B68] Martinez-Valdes E. Negro F. Laine C. M. Falla D. Mayer F. Farina D. (2017). Tracking motor units longitudinally across experimental sessions with high-density surface electromyography. J. Physiol. 595, 1479–1496. 10.1113/JP273662 28032343 PMC5330923

[B69] Mei Z. Grummer-Strawn L. M. Pietrobelli A. Goulding A. Goran M. I. Dietz W. H. (2002). Validity of body mass index compared with other body-composition screening indexes for the assessment of body fatness in children and adolescents. Am. J. Clin. Nutr. 75, 978–985. 10.1093/ajcn/75.6.978 12036802

[B70] Mizumura K. Taguchi T. (2024). Neurochemical mechanism of muscular pain: insight from the study on delayed onset muscle soreness. J. physiological Sci. JPS, 4. 10.1186/s12576-023-00896-y PMC10809664 38267849

[B71] Moritani T. deVries H. (1979). Neural factors versus hypertrophy in the time course of muscle strength gain. Am. J. Phys. Med. 58 (3), 115–130. Available at: https://pubmed.ncbi.nlm.nih.gov/453338/(Accessed: August 5, 2024). 453338

[B72] Newham D. J. (1988). The consequences of eccentric contractions and their relationship to delayed onset muscle pain. Eur. J. Appl. Physiol. 57, 353–359. 10.1007/BF00635995 3371343

[B73] Nuccio S. Del Vecchio A. Casolo A. Labanca L. Rocchi J. E. Felici F. (2021). Deficit in knee extension strength following anterior cruciate ligament reconstruction is explained by a reduced neural drive to the vasti muscles. J. Physiology 599, 5103–5120. 10.1113/JP282014 34605556

[B74] Nuzzo J. Barry B. K. Jones M. D. Gandevia S. C. Taylor J. L. (2017). Effects of four weeks of strength training on the corticomotoneuronal pathway. Med. Sci. Sports Exerc 49 (11), 2286–2296. 10.1249/MSS.0000000000001367 28692630

[B75] Oldfield R. C. (1971). The assessment and analysis of handedness: the Edinburgh inventory. Neuropsychologia 9 (1), 97–113. 10.1016/0028-3932(71)90067-4 5146491

[B76] Orssatto L. B. R. Rodrigues P. Mackay K. Blazevich A. J. Borg D. N. Souza T. R. d. (2023). Intrinsic motor neuron excitability is increased after resistance training in older adults. J. Neurophysiology 129 (3), 635–650. 10.1152/jn.00462.2022 36752407

[B77] Paillard T. (2020). Cross-education related to the ipsilateral limb activity on monopedal postural control of the contralateral limb: a review. Front. Physiology 11, 496. 10.3389/fphys.2020.00496 PMC7253698 32528312

[B78] Pasquet B. Carpentier A. Duchateau J. (2005). Change in muscle fascicle length influences the recruitment and discharge rate of motor units during isometric contractions. J. Neurophysiology 94 (5), 3126–3133. 10.1152/jn.00537.2005 16014788

[B79] Pearcey G. E. P. Alizedah S. Power K. E. Button D. C. (2021). Chronic resistance training: is it time to rethink the time course of neural contributions to strength gain? Eur. J. Appl. Physiology 121, 2413–2422. 10.1007/s00421-021-04730-4 34052876

[B97] Pelet D. C. S. Orsatti F. L. (2021). Effects of resistance training at different intensities of load on cross-education of muscle strength. Appl Physiol Nutr Metab. 46 (10), 1279–1289. 10.1139/apnm-2021-0088 33984253

[B80] Piasecki J. Škarabot J. Spillane P. Piasecki M. Ansdell P. (2024). Sex differences in neuromuscular aging: the role of sex hormones. Exerc. Sport Sci. Rev. 52, 54–62. 10.1249/JES.0000000000000335 38329342

[B81] Reif A. Wessner B. Haider P. Tschan H. Triska C. (2021). Strength performance across the oral contraceptive cycle of team sport athletes: a cross-sectional study. Front. Physiology 12, 658994. 10.3389/fphys.2021.658994 PMC8281678 34276392

[B82] Richmond S. B. Fling B. W. (2019). Transcallosal control of bilateral actions. Exerc. Sport Sci. Rev. 47 (4), 251–257. 10.1249/JES.0000000000000202 31525166

[B83] Roberts M. D. McCarthy J. J. Hornberger T. A. Phillips S. M. Mackey A. L. Nader G. A. (2023). Mechanisms of mechanical overload-induced skeletal muscle hypertrophy: current understanding and future directions. Physiol. Rev. NLM (Medline) 103, 2679–2757. 10.1152/physrev.00039.2022 PMC10625844 37382939

[B84] Ruddy K. L. Carson R. G. (2013). Neural pathways mediating cross education of motor function. Front. Hum. Neurosci. 7 (397), 397. 10.3389/fnhum.2013.00397 23908616 PMC3725409

[B85] Sale D. (1988). Neural adaptation to resistance training. Med. Sci. sports Exerc. 20 (5), 135–145. 10.1249/00005768-198810001-00009 3057313

[B86] Sato S. Yoshida R. Kiyono R. Yahata K. Yasaka K. Nosaka K. (2021). Cross-education and detraining effects of eccentric vs. concentric resistance training of the elbow flexors. BMC Sports Sci. Med. Rehabilitation 13 (1), 105. 10.1186/s13102-021-00298-w PMC8419922 34488881

[B87] Scripture E. W. Smith T. L. Brown E. M. (1894). On the education of muscular control and power. Stud. Yale Psychol. Lab. 2 (5).

[B88] Škarabot J. Brownstein C. G. Casolo A. Del Vecchio A. Ansdell P. (2021). The knowns and unknowns of neural adaptations to resistance training. Eur. J. Appl. Physiology 121, 675–685. 10.1007/s00421-020-04567-3 PMC7892509 33355714

[B89] Song J. S. Yamada Y. Kataoka R. Hammert W. B. Kang A. Loenneke J. P. (2024). Cross-education of muscular endurance: a scoping review. Sports Med. Springer Sci. Bus. Media Deutschl. GmbH 54, 1771–1783. 10.1007/s40279-024-02042-z PMC11258191 38758463

[B90] Stein R. B. Parmiggiani F. (1979). Optimal motor patterns for activating mammalian muscle. Brain Res. 175, 372–376. 10.1016/0006-8993(79)91019-9 226227

[B91] Tenan M. S. Peng Y. L. Hackney A. C. Griffin L. (2013). Menstrual cycle mediates vastus medialis and vastus medialis oblique muscle activity. Med. Sci. Sports Exerc. 45 (11), 2151–2157. 10.1249/MSS.0b013e318299a69d 23657168

[B92] Tracy B. L. Maluf K. S. Stephenson J. L. Hunter S. K. Enoka R. M. (2005). Variability of motor unit discharge and force fluctuations across a range of muscle forces in older adults. Muscle Nerve 32 (4), 533–540. 10.1002/mus.20392 15986419

[B93] Vila-Chã C. Falla D. (2016). Strength training, but not endurance training, reduces motor unit discharge rate variability. J. Electromyogr. Kinesiol. 26, 88–93. 10.1016/j.jelekin.2015.10.016 26586649

[B94] Weidauer L. Zwart M. B. Clapper J. Albert J. Vukovich M. Specker B. (2020). Neuromuscular performance changes throughout the menstrual cycle in physically active females. J. Musculoskelet. Neuronal Interact. 20 (3), 314–324. Available at: http://www.ismni.org. 32877968 PMC7493438

[B95] Wilkinson R. D. Mazzo M. R. Feeney D. F. (2023). Rethinking the statistical analysis of neuromechanical data. Exerc. Sport Sci. Rev. 51 (1), 43–50. 10.1249/JES.0000000000000308 36206407

[B96] Yu B. Zhang X. Cheng Y. Liu L. YanJiang Wang J. (2022). The effects of the biceps brachii and brachioradialis on elbow flexor muscle strength and spasticity in stroke patients. Neural Plast. 2022, 1295908. 10.1155/2022/1295908 35283993 PMC8906960

